# A Nice Thing for a Doctor

**Published:** 1878-08-20

**Authors:** 


### A NICE THING FOR A DOCTOR.

The place mentioned in our last number, as admirably suited for a hygiene home, is at a railroad village in upper Georgia, and is equally well suited for a stock or dairy farm, 125 acres, one-half in cultivation, with springs, orchard, dwelling, etc. Address Business Manager of this journal.

